# Mesoniviridae: a proposed new family in the order *Nidovirales* formed by a single species of mosquito-borne viruses

**DOI:** 10.1007/s00705-012-1295-x

**Published:** 2012-04-24

**Authors:** Chris Lauber, John Ziebuhr, Sandra Junglen, Christian Drosten, Florian Zirkel, Phan Thi Nga, Kouichi Morita, Eric J. Snijder, Alexander E. Gorbalenya

**Affiliations:** 1 Molecular Virology Laboratory, Department of Medical Microbiology, Leiden University Medical Center, Albinusdreef 2, E4-P, P.O. Box 9600, 2300 RC Leiden, The Netherlands; 2 Institute of Medical Virology, Justus Liebig University Giessen, Giessen, Germany; 3 Institute of Virology, University of Bonn Medical Center, Bonn, Germany; 4 Department of Virology, National Institute of Hygiene and Epidemiology, Hanoi, 100-000 Vietnam; 5 Department of Virology, Institute of Tropical Medicine, Global COE Program, Nagasaki University, Nagasaki, 852-8523 Japan

**Keywords:** Subgenomic RNAs, Ancestral State Reconstruction, Murine Hepatitis Virus, Yellow Head Virus, Taxonomic Proposal

## Abstract

Recently, two independent surveillance studies in Côte d’Ivoire and Vietnam, respectively, led to the discovery of two mosquito-borne viruses, Cavally virus and Nam Dinh virus, with genome and proteome properties typical for viruses of the order *Nidovirales*. Using a state-of-the-art approach, we show that the two insect nidoviruses are (i) sufficiently different from other nidoviruses to represent a new virus family, and (ii) related to each other closely enough to be placed in the same virus species. We propose to name this new family Mesoniviridae. *Meso* is derived from the Greek word “mesos” (in English “in the middle”) and refers to the distinctive genome size of these insect nidoviruses, which is intermediate between that of the families *Arteriviridae* and *Coronaviridae*, while *ni* is an abbreviation for “nido”. A taxonomic proposal to establish the new family Mesoniviridae, genus Alphamesonivirus, and species Alphamesonivirus 1 has been approved for consideration by the Executive Committee of the ICTV.

The order *Nidovirales* [1] includes positive-sense single-stranded RNA (ssRNA+) viruses of three families: *Arteriviridae* [2] (12.7–15.7-kb genomes; “small-sized nidoviruses”), *Coronaviridae* [3] and *Roniviridae* [4] (26.3–31.7 kb; the last two families are jointly referred to as “large-sized nidoviruses”) [5]. All other known ssRNA+ viruses have genome sizes below 20 kb. Recently, two closely related viruses, Cavally virus (CAVV) and Nam Dinh virus (NDiV), were discovered by two independent groups of researchers in Côte d’Ivoire in 2004 and in Vietnam in 2002, respectively [6, 7]. CAVV was isolated from various mosquito species belonging to the genera *Culex*, *Aedes*, *Anopheles* and *Uranotaenia* [7]. It was most frequently found in *Culex* species, especially *Culex nebulosus*. Except for *Culex quinquefasciatus*, which circulates worldwide, the other mosquito species are endemic to Africa. NDiV was isolated from *Culex vishnui*, which is endemic to Asia, and *Culex tritaeniorhynchus*, which circulates in Asia and Africa [6], and there are indications that it may infect more mosquito species (Nga, unpublished data). Analysis of abundance patterns of 39 CAVV isolates in different habitat types along an anthropogenic disturbance gradient has indicated an increase in virus prevalence from natural to modified habitat types [8]. A significantly higher prevalence was found especially in human settlements. Analysis of habitat-specific virus diversity and ancestral state reconstruction demonstrated an origin of CAVV in a pristine rainforest with subsequent spread into agriculture and human settlements [7]. Notably, it was shown for the first time that virus diversity decreased and prevalence increased during the process of emergence from a pristine rainforest habitat into surrounding areas of less host biodiversity due to anthropogenic modification [7]. Both viruses were propagated in *Aedes albopictus* cells and characterized using different techniques. A number of common properties place CAVV and NDiV in the order *Nidovirales*. These properties include (i) the genome organization with multiple open reading frames (ORFs), (ii) the predicted proteomes (Fig. 1), (iii) the production of enveloped, spherical virions, and (iv) the synthesis of genome-length and subgenome-length viral RNAs in infected cells [6, 7]. Particularly, the two viruses were found to encode key molecular markers characteristic of all nidoviruses: a 3C-like main protease (3CLpro, also known as Mpro) flanked by two transmembrane (tM) domains encoded in replicase ORF1a, as well as an RNA-dependent RNA polymerase (RdRp) and a combination of a Zn-binding module (Zm) fused with a superfamily 1 helicase (HEL1) encoded in ORF1b. As in other nidovirus genomes, ORFs 1a and 1b were found to overlap by a few nucleotides in both CAVV and NDiV. The ORF1a/1b overlap region includes a putative -1 ribosomal frameshift site (RFS) that is expected to direct the translation of ORF1b by a fraction of the ribosomes that start translation at the ORF1a initiation codon. Thus, a frameshift just upstream of the ORF1a termination codon mediates the production of a C-terminally extended polyprotein jointly encoded by ORF1a and ORF1b. Combined, these markers form the characteristic nidovirus constellation: tM-3CLpro-tM_RFS_RdRp_Zm-HEL1 (Fig. 1) [1, 5]. Likewise, virion proteins are encoded in ORFs that are located downstream of ORF1b and expressed from a set of subgenomic mRNAs. No similarities were found between the (putative) structural proteins of CAVV and NDiV and those of other nidoviruses [6, 7]. The most distinctive molecular characteristic of CAVV and NDiV, however, is the ~20-kb genome size, that is intermediate between the size ranges of small-sized and large-sized nidovirus genomes. Consequently, each of the two viruses has been proposed to prototype a new nidovirus family [6, 7].

**Fig. 1:**
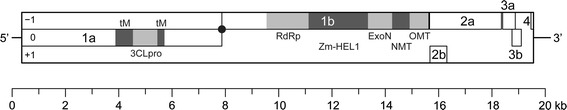
Genome organization of mesoniviruses. The coding and 5′- and 3′-untranslated regions of the genome are represented, respectively, by the outer rectangle and horizontal lines. ORFs are shown as open rectangles and are arranged in three reading frames (−1, 0, +1) relative to that of ORF1a. ORF1a- and ORF1b-encoded protein domains identified by bioinformatics analysis (see ref. [6]) are highlighted in grey. The predicted location of −1 ribosomal frameshift signals are indicated by a black dot. The genome organization is shown for NDiV but is virtually identical to that of CAVV except for the reading frame of some ORFs (see Table 1)

In this study, we compared the genomes of CAVV (GenBank accession number HM746600) and NDiV (GenBank accession number DQ458789) to assess their relationship and use this insight for taxonomic classification of these viruses. To date, only very limited biological information is available for CAVV and NDiV (see above), and in general, biological properties may be affected profoundly by a few changes in the genome. In view of these considerations and in line with the accepted taxonomic approach to viruses of the family *Coronaviridae* [3], comparative sequence analysis was considered the most reliable basis for classification. The overall similarity between the CAVV and NDiV genomes was found to be strikingly high: nearly identical sizes (20,187 and 20,192 nt, respectively), conservation of ORFs with sequence identities ranging from 87.8 to 96.1% at the amino acid level and from 88.3 to 93.7% at the nucleotide level (Table 1). Given this high similarity, prior assignments of domains and genetic signals were cross-checked to produce a unified description.

**Table 1:** Comparison of ORFs in the genome of NDiV and CAVV

	Length [nt]		Frame^a^		Identity [%]^b^		Predicted protein
	NDiV	CAVV	NDiV	CAVV	nt	aa	
ORF1a	7509	7497	0	0	88.3	90.0	Polyprotein 1a
ORF1b	7587	7587	−1	−1	92.6	96.1	1b part of polyprotein 1ab
ORF2a	2697	2700	−1	−1	90.7	87.5	Spike
ORF2b	636	642	+1	+1	88.8	90.2	Nucleocapsid
ORF3a	474	474	−1	+1	91.1	93.0	Membrane
ORF3b	348	348	0	−1	93.7	90.5	Membrane
ORF4	135	147	+1	−1	89.9	87.8	Unknown

ORF designations according to Table 2 in ref. [7]

^a^Reading frame relative to that of ORF1a

^b^Pairwise nucleotide (nt) and amino acid (aa) sequence identity between NDiV and CAVV

There was complete agreement between the two studies [6, 7] on the mapping of all nidovirus-wide conserved domains in CAVV and NDiV, as well as on the identification of GGAUUUU as a plausible slippery sequence in RFS (see above). Additionally, our analysis showed that the NDiV-based assignment [6] of 3′-to-5′ exoribonuclease (ExoN) and 2′-O-methyltransferase (OMT), two replicative domains characteristic for large-sized nidoviruses [5], and N7-methyltransferase (NMT) [9] in ORF1b extends to CAVV. Likewise, CAVV may lack a uridylate-specific endonuclease (NendoU), as has previously been observed for NDiV [6]. The synthesis of subgenomic RNAs from which ORFs 2a to 4 are predicted to be expressed appears to be controlled by transcription-regulating sequences (TRSs) [10–12] identified upstream of ORF2a/2b, ORF3a and ORF4 (collectively designated as body TRSs). Other putative TRSs were identified downstream of the leader region located at the 5′-end of the viral genome [6, 7]. Unique among nidoviruses, NDiV and CAVV may use different leader TRSs during the synthesis of different subgenomic RNAs, although further analysis is required to clarify the basis for some discrepancies between the TRS assignment in NDiV and CAVV. Also, it remains to be shown why the high sequence conservation of virion proteins of the two viruses (Table 1) was not manifested in the morphology observed upon EM analysis of virus particles [6, 7]. In this respect, it may be relevant that Zirkel et al. [7] noticed two types of particles in CAVV-infected cells, one of which carried club-shaped surface projections compatible with viral glycoproteins. This latter type of particles was also observed in infected cell culture supernatant. Ultimately, the origin of the particles of both types, and their relationship to the particles isolated from the medium of NDiV-infected C6/36 cells by Nga et al. [6] should be revealed by future research efforts.

Furthermore, we evaluated the phylogenetic position of CAVV and NDiV in relation to other nidoviruses. We conducted a phylogenetic analysis as described in ref. [6]. The study indicates that CAVV and NDiV consistently, albeit very distantly, cluster with viruses of the family *Roniviridae*, the only other known nidoviruses infecting invertebrates (Fig. 2). Quantitatively, this Bayesian posterior probability phylogeny illustrates that CAVV and NDiV form a deeply rooted lineage in the nidovirus tree with an evolutionary divergence from other nidoviruses comparable to that separating viruses of the families *Coronaviridae and Roniviridae* (Fig. 2). Together, these characteristics of CAVV and NDiV (insect host, intermediate genome size, deeply rooted phylogenetic lineage) provide a compelling basis for the creation of a new nidovirus family. We propose to name this new family Mesoniviridae, where *meso* is derived from the Greek word “mesos” (in English “middle” or “in the middle”) and refers to a key distinctive characteristic of these viruses, namely their intermediate-sized genomes. The second component of the acronym, *ni*, refers to *ni*doviruses, as has been done previously for ro*ni*viruses [13] and bafi*ni*viruses [14].

**Fig. 2:**
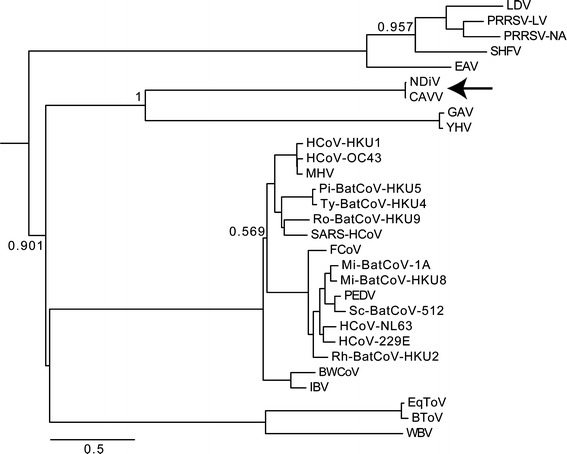
Phylogenetic position of CAVV and NDiV. To infer phylogenetic relationships of Nam Dinh virus isolate 02VN178 (NDiV), Cavally virus isolate C79 (CAVV) (arrow) and other nidoviruses, a partially constrained tree was calculated using a concatenated alignment of the three nidovirus-wide conserved domains and a set of viruses representing currently recognized species. The alignment was produced with Muscle version 3.52 [17] in the Viralis platform [18], and the phylogenetic analysis was performed using BEAST version 1.4.7 [19]. For further details, see ref. [6]. Numbers indicate posterior probability support values (on a scale from 0 to 1); all internal nodes for which no support value is provided have been fixed in the analysis based on prior analyses of nidovirus subsets (data not shown). The scale bars represent the average number of substitutions per amino acid position. The tree was rooted on the arterivirus branch. Virus names and GenBank/Refseq accession numbers: lactate dehydrogenase-elevating virus (LDV; U15146), porcine respiratory and reproductive syndrome virus European type (PRRSV-LV; M96262), porcine respiratory and reproductive syndrome virus North American type (PRRSV-NA; AF176348), simian hemorrhagic fever virus (SHFV; NC_003092), equine arteritis virus (EAV; AY349167), Nam Dinh virus (NDiV; DQ458789), Cavally virus (CAVV; HM746600), gill-associated virus (GAV; AF227196), yellow head virus (YHV; EU487200), human coronavirus HKU1 (HCoV-HKU1; AY884001), human coronavirus OC43 (HCoV-OC43; AY585228), mouse hepatitis virus (MHV; AY700211), Pipistrellus bat coronavirus HKU5 (Pi-BatCoV-HKU5; EF065509), Tylonycteris bat coronavirus HKU4 (Ty-BatCoV-HKU4; EF065505), Rousettus bat coronavirus HKU9 (Ro-BatCoV-HKU9; EF065513), SARS coronavirus (SARS-HCoV; AY345988), feline coronavirus (FCoV; NC_007025), Miniopterus bat coronavirus 1A (Mi-BatCoV-1A; NC_010437), Miniopterus bat coronavirus HKU8 (Mi-BatCoV-HKU8; NC_010438), porcine epidemic diarrhoea virus (PEDV; NC_003436), Scotophilus bat coronavirus 512 (Sc-BatCoV-512; DQ648858), human coronavirus NL63 (HCoV-NL63; DQ445911), human coronavirus 229E (HCoV-229E; NC_002645), Rhinolophus bat coronavirus HKU2 (Rh-BatCoV-HKU2; NC_009988), beluga whale coronavirus SW1 (BWCoV; EU111742), avian infectious bronchitis virus (IBV; NC_001451), equine torovirus (EToV; X52374), bovine torovirus (BToV; NC_007447), white bream virus (WBV; NC_008516)

Next, we sought to establish species demarcation criteria to decide whether CAVV and NDiV prototype separate species or belong to a single species. Commonly, this question cannot be answered (reliably) on the basis of only two full genome sequences and otherwise very limited biological data. To solve this dilemma, we exploited information available for other nidoviruses in our analysis. In order to evaluate the genetic similarity between CAVV and NDiV in the context of sequence divergence of lineages representing previously established nidovirus species, we applied a state-of-the-art framework for a genetics-based classification [15]. This recently introduced classification approach has been shown to recover and refine the taxonomy of picornaviruses [16], and it was also used to revise the taxonomy of coronaviruses extensively (Lauber & Gorbalenya, in preparation) [3]. In addition to CAVV and NDiV, a representative set of 152 large-sized nidoviruses was included in the analysis. Two sets of proteins were used: the first included proteins conserved in all nidoviruses (3CLpro, RdRp, HEL1) (dataset D1), while the second set additionally included ExoN and OMT, which are conserved in large-sized nidoviruses and CAVV/NDiV (dataset D2). For both datasets a concatenated, multiple amino acid alignment was produced, which formed the basis for compiling pairwise evolutionary distances (PEDs) between all pairs of viruses (Fig. 3ab; for details see ref. [15]). It was found that the PED separating CAVV and NDiV is within the range of intra-species virus divergence in the families *Coronaviridae* and *Roniviridae* for both datasets (Fig. 3cd). Specifically, CAVV and NDiV show a distance (0.016 and 0.029 for D1 and D2, respectively) that is below the genetic divergence of members of several established nidovirus species (maximum of 0.032 and 0.037 for D1 and D2, respectively). For both datasets, these viruses include gill-associated virus and yellow head virus (species *Gill-associated virus*, family *Roniviridae*) [4] and the coronaviruses feline coronavirus, transmissible gastroenteritis virus, and porcine respiratory coronavirus (species *Alphacoronavirus 1*), IBV (species *Avian coronavirus*), murine hepatitis virus (species *Murine coronavirus*), and Rousettus bat coronavirus HKU9 (species *Rousettus bat coronavirus HKU9*) [3]. For the dataset comprising the three nidovirus-wide conserved proteins (Fig. 3ac), Miniopterus bat coronavirus 1 also showed a maximum genetic divergence exceeding that of the CAVV-NDiV pair. Together, these observations show that CAVV and NDiV belong to the same species, representing a single genus in the family. We propose to name this genus Alphamesonivirus and the species Alphamesonivirus 1, thereby following a naming convention recently applied to the subfamily *Coronavirinae* [3], which is expected to facilitate the accommodation of future expansions of the family. A taxonomic proposal for family, genus, and species recognition has been available on-line at the ICTV website (http://talk.ictvonline.org/files/proposals/taxonomy_proposals_invertebrate1/m/default.aspx) since August 2011. It has been approved by the chairs of the ICTV *Arteriviridae*, *Coronaviridae*, and *Roniviridae* Study Groups and the Executive Committee of the ICTV, and will be considered again at the next EC-ICTV meeting, to be held in Leuven, Belgium, in July 2012.

**Fig. 3:**
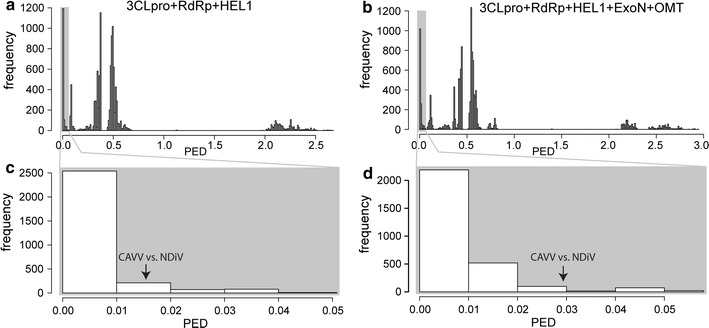
Evolutionary distance between CAVV and NDiV in relation to intra-species genetic divergence in large-sized nidoviruses. Multiple amino acid alignments for 154 nidoviruses with large genomes (all major nidovirus lineages except arteriviruses) comprising three nidovirus-wide conserved protein domains (**a**, **c**) or five domains conserved in all large-sized nidoviruses (**b**, **d**) were used to compile pairwise evolutionary distances (PEDs) between all virus pairs. These distances are shown as frequency distributions (**a**, **b**), and zoom-ins on small distances are provided (**c**, **d**). The PED between CAVV and NDiV (indicated by the arrow) is well within the intra-species distance range of other nidoviruses. Several currently recognized nidovirus species show a maximum genetic divergence larger than that of the CAVV-NDiV pair (see text)

The recognition of CAVV and NDiV as a single virus species can be contrasted with the detection of these viruses in many mosquito host species and their spread to different continents (Africa and Asia, respectively) [6, 7]. The underlying mechanisms of this broad dispersal are unknown but might include the crossing of the host species barrier rather than virus-host cospeciation. Further research, including the characterization of biological properties of CAVV and NDiV and the extension of surveillance studies to other regions of the world, is needed to understand the ecology, host tropism and medical and/or economic relevance of mesoniviruses.
